# Angiotensin II-induced hypertension does not exacerbate α-SYNUCLEIN pathology in a mouse model of Parkinson’s disease

**DOI:** 10.3389/fimmu.2026.1894579

**Published:** 2026-09-22

**Authors:** Ignacio Silva-Llanes, Daniel Flores-Téllez, Wilfrido J. Arrua, Naoual B. El Jouhari, Eduardo Oliver, Ana Martínez, Ana M. Briones, Isabel Lastres-Becker

**Affiliations:** 1 Instituto de Investigaciones Biomédicas “Sols-Morreale” CSIC-UAM, Madrid, Spain; 2 Instituto de Investigación Sanitaria La Paz (IdiPaz), Madrid, Spain; 3 Dpto. Farmacología, Facultad de Medicina, Universidad Autónoma de Madrid, Madrid, Spain; 4 Centro de Investigación Biomédica en Red Enfermedades Cardiovasculares, CIBERCV, Madrid, Spain; 5 Centro de Investigaciones Biológicas Margarita Salas (CSIC), Madrid, Spain; 6 Centro Nacional de Investigaciones Cardiovasculares (CNIC), Madrid, Spain; 7 Centro de Investigación Biomédica en Red, Área Enfermedades Neurodegenerativas, CIBERNED, Instituto de Salud Carlos III, Madrid, Spain

**Keywords:** α-SYNUCLEIN, angiotensin-II, gasdermin, hypertension, neurodegeneration, neuroinflammation, Parkinson’s disease

## Abstract

Parkinson’s disease (PD) is characterized by progressive degeneration of nigrostriatal dopaminergic neurons accompanied by chronic neuroinflammation and α-SYNUCLEIN (α-SYN) aggregation. Increasing evidence suggests that cardiovascular comorbidities, including arterial hypertension, may contribute to neurodegenerative processes through vascular dysfunction and sustained inflammatory signaling. However, whether hypertension directly exacerbates α-SYN-induced neurodegeneration and inflammasome activation remains unclear. Here, we investigated the impact of chronic Angiotensin II (AngII)-induced hypertension in an adeno-associated viral vector (AAV)-based mouse model of PD driven by unilateral overexpression of human α-SYN in the *Substantia Nigra* (SN). Chronic AngII infusion produced a robust hypertensive phenotype but did not aggravate α-SYN-induced loss of tyrosine hydroxylase-positive dopaminergic neurons. Similarly, hypertension failed to modify α-SYN-driven astrogliosis, microgliosis, or microglial morphological activation. Molecular analyses revealed that α-SYN overexpression markedly increased the expression of inflammatory mediators and pyroptosis-related genes, including *Nlrp3*, *Asc*, *Casp1*, *Il1b*, *Il18rap*, and *Gsdmd*. Nevertheless, hypertension did not potentiate these responses. Immunofluorescence analyses further demonstrated that GSDMD expression was predominantly localized in IBA1-positive microglial cells and was significantly elevated following α-SYN overexpression independently of hypertensive status. Collectively, these findings indicate that chronic AngII-induced hypertension is insufficient to exacerbate early α-SYN-mediated neurodegeneration, gliosis, or inflammasome-associated pyroptotic pathways in the SN. Our study suggests that α-SYN pathology itself constitutes the dominant driver of neuroinflammatory activation in this experimental paradigm and that additional vascular or aging-related mechanisms may be required for hypertension to significantly influence PD progression.

## 1 Introduction

Parkinson’s disease (PD) is the second most common neurodegenerative disorder and is characterized by progressive degeneration of dopaminergic neurons in the SN pars compacta together with the accumulation of misfolded α-SYN in Lewy bodies and Lewy neurites (1–3). Beyond neuronal dysfunction, a substantial body of evidence supports a central contribution of neuroinflammatory mechanisms to PD pathogenesis, particularly through the activation of astrocytes and microglia that sustain chronic inflammatory responses and neuronal injury (4–6). Microglial activation is now recognized as a major driver of PD progression. Pathological α-SYN species can activate innate immune signaling pathways, including Nuclear factor kappa-light-chain-enhancer of activated B cells (NF-κB)-dependent transcriptional programs and inflammasome activation, leading to the release of pro-inflammatory cytokines and reactive oxygen species. Among these pathways, the NLRP3 inflammasome has emerged as a key mediator linking α-SYN aggregation to neurodegeneration (7–12). Activation of the NLRP3/ASC/CASPASE-1 axis promotes the maturation of IL-1β and IL-18 and induces GASDERMIN D (GSDMD)-dependent pyroptosis, a highly inflammatory form of programmed cell death increasingly implicated in PD pathology.

In parallel with neuroinflammatory processes, epidemiological studies indicate that vascular and metabolic disorders may influence the onset and progression of neurodegenerative diseases. Arterial hypertension, the most widespread cardiovascular diseases globally, has been linked to cerebrovascular impairment, oxidative stress, disruption of the blood-brain barrier, and persistent low-grade neuroinflammation within the central nervous system (13–18). Experimental evidence further demonstrates that hypertension can promote neuroinflammatory responses and neuronal vulnerability, suggesting that systemic vascular dysfunction may sensitize the brain to neurodegenerative insults (14, 19).

Several clinical studies have proposed a relationship between hypertension and PD, although the nature of this association remains controversial. Meta-analyses have suggested that hypertension may increase the risk of PD or influence disease severity and progression (20, 21). Moreover, a bidirectional relationship between dopamine and local renin angiotensin aldosterone system (RAAS), which is one of the major contributors to hypertension, has been observed in PD which has led to the proposal that therapeutic strategies using angiotensin type I (AT1) receptor blockers and/or activation of the protective axis (AT2, Mas receptors), may be neuroprotective for individuals with a high risk of developing PD or in prodromal stages of PD to reduce progression of the disease (22). In addition, cardiovascular dysautonomia and altered blood pressure regulation are frequent non-motor manifestations in PD patients (23, 24). However, despite growing clinical evidence linking vascular dysfunction and PD, the mechanistic interactions between chronic hypertension and α-SYN-driven neurodegeneration remain poorly understood.

Given the established contribution of hypertension to systemic and cerebral inflammation, we hypothesized that chronic arterial hypertension could potentiate α-SYN-induced neuroinflammatory and neurodegenerative processes in PD. To address this question, we combined chronic Angiotensin II (AngII)-induced hypertension with adeno-associated viral vector (AAV)-mediated overexpression of human α-SYN in the SN of mice. We specifically investigated whether hypertension exacerbates dopaminergic neuronal loss, gliosis, inflammatory signaling, and activation of pyroptosis-related pathways associated with inflammasome activation.

## 2 Methods

### 2.1 Angiotensin II hypertension mouse model

The mice were bred and housed (three to four mice per cage) in temperature-controlled cages (~23°C) under a 12/12 h light/dark cycle with free access to water and standard chow in the Universidad Autónoma of Madrid Animal Core. Subcutaneous osmotic minipumps (Model 2004 Durect Corp, Cupertino, CA) containing AngII (1.44 mg/Kg/day; Sigma-Aldrich) were implanted in the back of anesthetized 6-month-old C57BL/6 mice (isoflurane 1.5%) and left for 21 days (n=15). For the implantation of osmotic minipumps, the mice were anaesthetized with isoflurane inhalation (1.5%) (25). Anesthetic depth was confirmed by loss of blink reflex and/or lack of response to tail pinch. The procedure took approximately 15 min per mouse. Recovery after surgical procedures was performed using aseptic techniques in a dedicated approved surgical area. Analgesics (buprenorphine, 0.05 mg/Kg−1, s.c.) were administered immediately after surgery to prevent discomfort. The animals were kept warm in a heating pad until awake after surgery and observed carefully by the investigators throughout the postsurgery period.

Weekly weight control was performed from the beginning of the experiment and arterial pressure was measured by tail-cuff plethysmography (Visitech Systems^®^ BP-2000-M-4 system, USA) one week before AngII minipumps implantation (**Figure 1A**). To minimize errors, animals were trained for one week prior to blood pressure measurements. At least six individual observations were performed at the same time of the day and averaged for each animal. Every effort was taken to minimize the number of animals used and their suffering. To estimate the appropriated sample size, we employed G*Power (Heinrich Heine Universität Düsseldorf).

**Figure 1:**
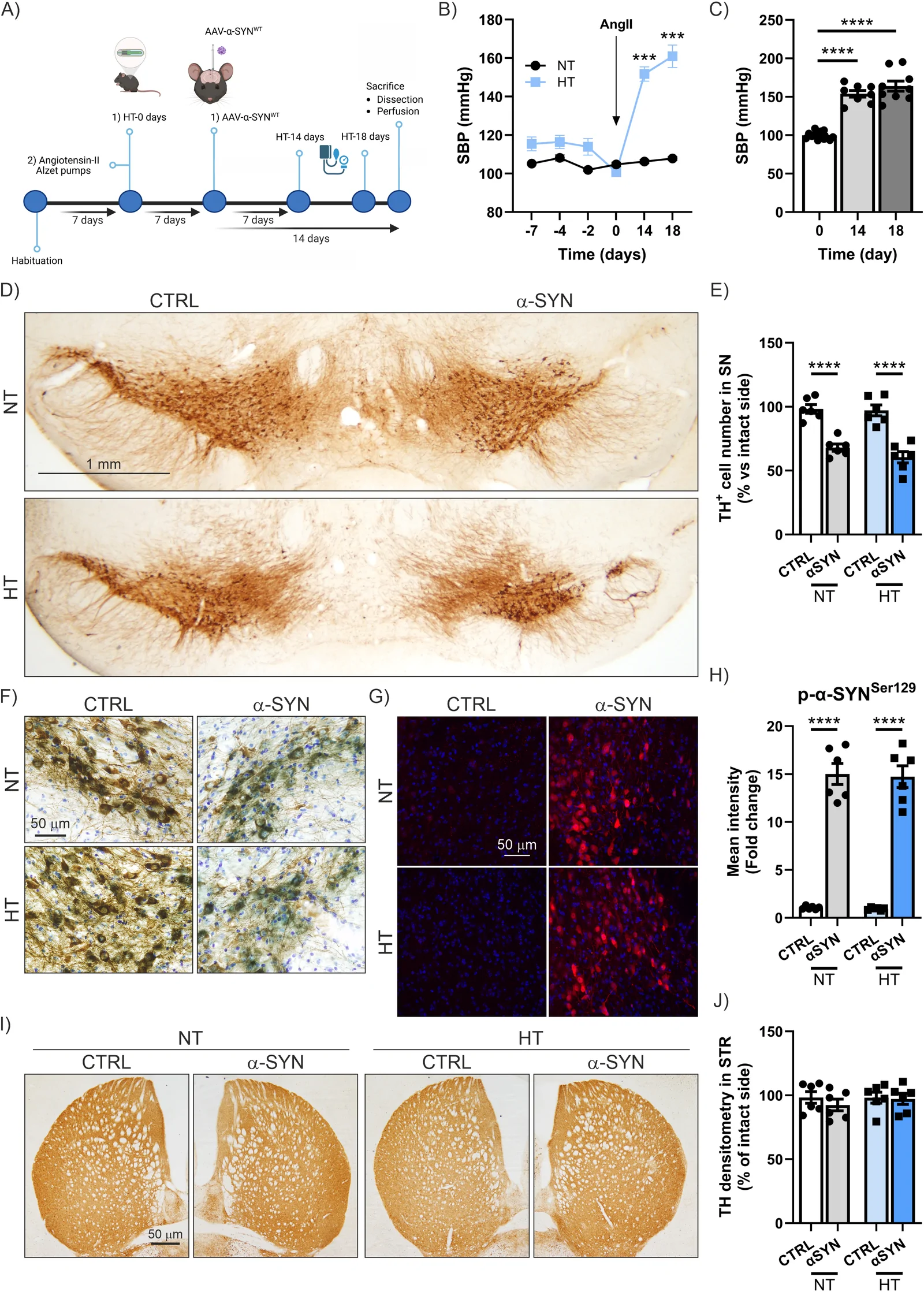
Arterial hypertension does not have an effect in α-SYN-induced neurodegeneration in a mouse model of PD. **(A)** Experimental design of the study. After 7 days of habituation, blood pressure was measured in the mice (HT-0), and subsequently, Alzet pumps were implanted subcutaneously for the release of AngII. Seven days later, stereotaxic surgeries were performed for the delivery of AAV vectors to induce unilateral overexpression of α-SYN in the SN. At 7 and 11 days after the stereotaxic surgery, blood pressure was measured (HT-14 and HT-18, respectively). After 14 days of α-SYN overexpression, the animals were sacrificed, and tissues were collected for subsequent analysis. **(B)** Comparison of systolic blood pressure (SBP, mmHg) between control mice (without Alzet pump implantation; gray) and mice implanted with AngII-filled Alzet pumps (blue). **(C)** Measure of the SBP (mmHG), at HT-0, HT-14 and HT-18 stages. **(D)** Representative images of TH immunohistochemistry in 30-µm sections of the SN (control and injected sides). **(E)** Stereological counting of TH^+^ nigral dopaminergic neurons at the α-SYN-overexpressing side of the SN (% vs the control side) of hypertensive (HT) mice and normotensive (NT) control mice. **(F)** Representative images showing the loss of TH expression of DAergic neurons after TH immunohistochemistry and Nissl staining. **(G)** Representative images of immunofluorescence of p-α-SYN^Ser129^ (red) in 30-µm coronal sections of SN. **(H)** Quantification of p- α-SYN^Ser129^ fluorescence intensity. **(I)** Representative images of TH immunohistochemistry in 30-µm sections of the striatum (control and injected sides). **(J)** Densitometric quantification of TH^+^ dopaminergic terminals in the striatum. Bars represent the mean of 5–6 samples ± SEM. The asterisks represent the difference in significance: ***p<0.001, ****p<0.0001 comparing each group with the control side from NT mice according to two-way ANOVA followed by Bonferroni post-test. Comparisons without asterisks indicate no statistically significant differences (p > 0.05).

### 2.2 Randomization and blinding

Animals were randomized for treatment, and data collection and evaluation of all experiments were conducted blind to group identity. Data and statistical analyses were performed in accordance with recommended guidelines for experimental design and analysis in pharmacology (26).

### 2.3 Animal model of PD and stereotaxic injections

Each experimental group consisted of n=11 or 15. Viral vector injections were performed under ketamine/xylazine anesthesia (8 mg/kg ketamine and 1.2 mg/kg xylazine) on adult mice. Surgery was performed using a stereotaxic frame (Stoelting, Wood Dale, IL, USA) and a 5 µl Hamilton syringe fitted with a pulled glass capillary tube (outer diameter of 60–80 µm). Recombinant adeno-associated viral vectors of serotype 9, which express the human protein α-SYN under control of the synapsin 1 gene promoter (AAV-α-SYN), were injected in the right SN as described elsewhere (27–29). Briefly, 2 μl of AAV suspension containing 2×10E12 vp/ml were injected at the stereotaxic coordinates -2.8 mm posterior, -1.4 mm lateral, and -4.5 mm ventral relative to bregma, and the left SN was used as a control. A control AAV9 lacking α-SYN but expressing enhanced green fluorescent protein (EGFP) (AAV9-EGFP) did not have any significant effect on neuron viability, and a very low effect on gliosis and inflammation (statistically not significant) (data not shown). All experiments were performed in a P2 biosafety facility and by certified researchers according to regional, national, and European regulations concerning animal welfare and animal experimentation, and were authorized by the Ethics Committee for Research of the Universidad Autónoma de Madrid with Ref PROEX 152.1/24.

### 2.4 Behavioral test

The elevated body swing test is a simple and easy behavioral test that only requires handling the animal by its tail and recording the direction of swings made by the animal for a certain period (30, 31). Briefly, the animal was held in the vertical axis and a swing was recorded whenever the animal moved its head out of the vertical axis to either side. Before attempting another swing, the animal must return to the vertical position for the next swing to be counted. Each experimental group comprised 11–16 animals and the test was performed the last day, just before sacrifice.

### 2.5 Analysis of mRNA levels by quantitative real-time PCR

Total RNA extraction from ventral mid brain (VMB) samples, reverse transcription, and analysis of mRNA levels by quantitative real time PCR (q-RT-PCR) was performed as described in a previous article (32). One microgram of RNA from each sample was treated with DNase (Invitrogen) and reverse-transcribed using high-capacity RNA-to-cDNA Master Mix (Applied Biosystem). Primer sequences are presented in **Supplementary Table 1**. Data analysis was based on the 2^-ΔΔCT^ method with normalization of the raw data by the geometric mean of the housekeeping genes *Actb*, *Gapdh*, and *Tbp* (Applied Biosystems). All PCRs were performed in triplicate by using QuantStudio3 from Applied Biosystems.

### 2.6 Immunohistochemistry on mouse tissues

Mouse brain tissue was sectioned at 30 µm on a cryostat (Leica CM 1950) and stained as free-floating sections with Netwell baskets. As previously described (32), a standard avidin-biotin immunohistochemical protocol was used. Primary and secondary antibodies are described in **Supplementary Table 2**. Briefly, the primary antibody used was anti-human α-SYN or TH, for 24 h at RT. After washing, the sections were first incubated with the secondary biotinylated antibody and then with the ABC kit system to increase sensitivity and developed using 3,3-diaminobenzidine (DAB). Finally, tissues were dehydrated in ethanol and cleared in xylene. Sections were mounted with DEPEX and coverslipped. The immunohistochemistry images were captured using a directed Axiophot microscope (Zeiss) with transmitted light and epifluorescence. It has a camera with DP70 color with a DP Controller image capture system. The objectives that we used were: ACHROPLAN 4x and Plan-NEOFLUAR 10x/0.3.

### 2.7 Stereological analysis of TH^+^ neurons

Unbiased stereological estimation of tyrosine hydroxylase–positive (TH^+^) neurons in the SNpc was performed using Fiji software. Coronal brain sections (30 µm thick) encompassing the entire SNpc were collected in a systematic-random manner (every eighth section) and processed for TH immunohistochemistry. Only neurons with a clearly visible nucleus within the counting frame were included. SN, excluding the ventral tegmental area, was delineated in low magnification (5× objective), and a point grid was overlaid onto each section. Cell counting was performed as previously described by (27–29). The error coefficient attributable to the sampling was calculated according to (33), and values ≤ 0.10 were accepted (n = 5–6 animals per experimental group). Results were expressed as the total estimated number of TH^+^ neurons in the SNpc per hemisphere.

### 2.8 Nissl staining

After the immunostaining of TH and its subsequent stereological analysis, the coverslips were removed with xylene for 48 hours and the tissues were rehydrated through decreasing concentrations of alcohol. Subsequently, sections were incubated in Nissl’s solution (0.1% Cresyl Violet (Sigma), 2.5 ml acetic acid 10%) at room temperature for 15 min, rinsed in deionized water for 5 min, dehydrated in graded alcohols, cleared in xylene, and coverslipped again. Images were captured using a directed Axiophot microscope (Zeiss) with transmitted light and epifluorescence. It has a camera with DP70 color with a DP Controller image capture system. The objective that we used was Plan-NEOFLUAR 63x/1.25 oil.

### 2.9 Densitometric analysis of TH

Densitometric analysis of TH and immunostaining of the striatum were performed using Fiji software. Images were digitized through an Axiophot microscope connected to a digital camera (Olympus DP70) and analyzed in a blind fashion. Measurements were carried out throughout striatum. The average O.D. in the striatum was calculated after background subtraction. To compensate for small differences in absolute levels of stainings between sections, measurements from the lesion side were expressed as a percentage of the values from the contralateral side. (n = 6 animals per experimental group).

### 2.10 Immunofluorescence on mouse tissues

Immunofluorescence assays were performed on 30 µm-thick coronal brain sections at the SN. The protocol followed was previously described (34). Primary and secondary antibodies are described in **Supplementary Table 2**. Images were taken on the Leica Stellaris 8 TauSTED confocal microscope (SEMOC, Instituto de Investigaciones Biomédicas “Sols-Morreale”).

### 2.11 Stereological analysis of microgliosis and astrogliosis

Cell counts were performed using Fiji Software (http://fiji.sc/Fiji). For each mouse, 2–3 VMB sections were used for analysis (n=4–6 animals per experimental group) (35). The fluorescence values of each channel in each pixel of the image were represented on the same graph to obtain an intensity profile that reflected the colocalization of the proteins of interest (GraphPad Prism 10, San Diego, CA).

### 2.12 Morphological analysis of microglial cells

To analyze the cell size and the morphological structure of IBA1^+^ cells with Fiji software, we followed the previously described protocol (36). Briefly, the images were transformed into 8-bit format, and the threshold was adjusted to delimit the perimeter of the cells. *Unsharp Mask* and *Despeckle* tools were used to sharpen the images and remove unspecific spots in the background, respectively. With the *Analyze Particles* tool, the areas of each individual cell were determined to calculate the average cell size and circularity, a parameter representing the degree of roundness of cells. The same images were used to determine the number of branches and the average length of the branches with the *Skeletonize* and *Analyze skeleton* tools. (n=5–6 animals per experimental group).

### 2.13 Statistical analysis

Data are presented as mean ± SEM (Standard Error of the Mean). To confirm which statistical test had to be used, we employed GraphPad InStat 3 including the analysis of the data to a normal distribution via the Kolmogorov–Smirnov test. Furthermore, statistical assessments of differences between groups were analyzed (GraphPad Prism 5, San Diego, CA). Unpaired Student’s t-tests or two-way ANOVA with *post hoc* Bonferroni were used as appropriate.

### 2.14 Data availability

The data that supports the findings of this study is available by contacting the corresponding author on reasonable request.

## 3 Results

### 3.1 Hypertension does not potentiate α-SYN-induced nigral degeneration

To determine whether pre-existing hypertension modifies α-SYN-induced neurodegeneration, mice were first habituated to handling and blood pressure measurements for seven consecutive days before implantation of Alzet osmotic pumps, thereby reducing potential measurement-related artifacts (**Figures 1A, B**). Control groups did not undergo osmotic pump implantation, as previous work from our group has demonstrated that the osmotic pump with vehicle itself does not induce changes in blood pressure (37). Baseline blood pressure was recorded on day 0, immediately before implantation of pumps delivering Angiotensin II (AngII). Seven days later, animals underwent stereotaxic unilateral injection of an adeno-associated viral vector encoding human wild-type *SNCA* (AAV-α-SYN), driven by the neuron-specific SYNAPSIN-1 promoter. Blood pressure was reassessed 14 days after pump implantation, corresponding to 7 days after viral injection. At this stage, mean arterial pressure had risen to approximately 153,96 mmHg, representing a significant increase relative to baseline values and confirming the development of hypertension (**Figure 1C**). This elevation progressed further, reaching a mean of approximately 164,03 mmHg by day 18. Animals were sacrificed two days after the final blood pressure measurement. Blood pressure of control mice remained stable during the whole experiment (**Figure 1B**).

Correct stereotaxic targeting and comparable expression of AAV-α-SYN were verified across experimental groups, and animals lacking appropriate α-SYN expression were excluded from subsequent analyses. Quantification of tyrosine hydroxylase (TH)-positive neurons in the substantia nigra (SN) revealed a reduction on the injected side relative to the contralateral hemisphere, with an average loss of approximately 30% (**Figures 1D, E**). Notably, the hypertensive condition did not significantly aggravate α-SYN-induced degeneration of dopaminergic neurons. Nissl counterstaining further indicated that α-SYN overexpression not only reduced the number of TH-positive neurons but also diminished TH immunoreactivity in surviving cells (**Figure 1F**). This effect was attributable to α-SYN itself, since injection of a control AAV expressing EGFP did not modify TH-positive cell numbers relative to the non-injected side (data not shown). Additionally, we assessed α-SYN phosphorylation at Ser129, a well-established predictive marker of protein aggregation (38, 39). As shown in **Figures 1G, H**, α-SYN overexpression resulted in markedly increased Ser129 phosphorylation compared with the control side. These phosphorylation levels were not affected by hypertension, as no statistically significant differences were observed. Because neurons of the SN project to the striatum, TH immunoreactivity was also examined in striatal sections. No detectable differences in striatal TH density were observed two weeks after α-SYN overexpression (**Figures 1I, J**). Accordingly, subsequent analyses were focused on the SN. Overall, these results indicate that, within this experimental paradigm, hypertension induced by chronic AngII infusion does not synergize with α-SYN overexpression to exacerbate nigral dopaminergic neurodegeneration.

### 3.2 Hypertension does not alter α-SYN-induced astrogliosis in the SN

Hypertension has been described as generating a chronic inflammatory state that may exacerbate the neuroinflammation already present in the brains of PD patients, thereby accelerating the degeneration of dopaminergic neurons in the SN (14, 20, 40). Therefore, the next step was to determine whether the astrogliosis induced by α-SYN overexpression was modified by hypertension. Immunofluorescence analysis against GFAP (**Figure 2A**), an astrocyte marker, revealed that hypertension did not alter the astrogliosis induced by α-SYN overexpression, as no statistically significance differences were observed either in the number of GFAP^+^ cells (**Figure 2B**) or in *Gfap* mRNA levels measured by qPCR (**Figure 2C**). The observed astrogliosis was attributable to α-SYN overexpression rather than to the AAV injection itself, as no astrogliosis was detected following injection of AAV-EGFP (data not shown).Taken together, our data suggest that hypertension does not modify the astrogliosis induced by α-SYN overexpression.

**Figure 2:**
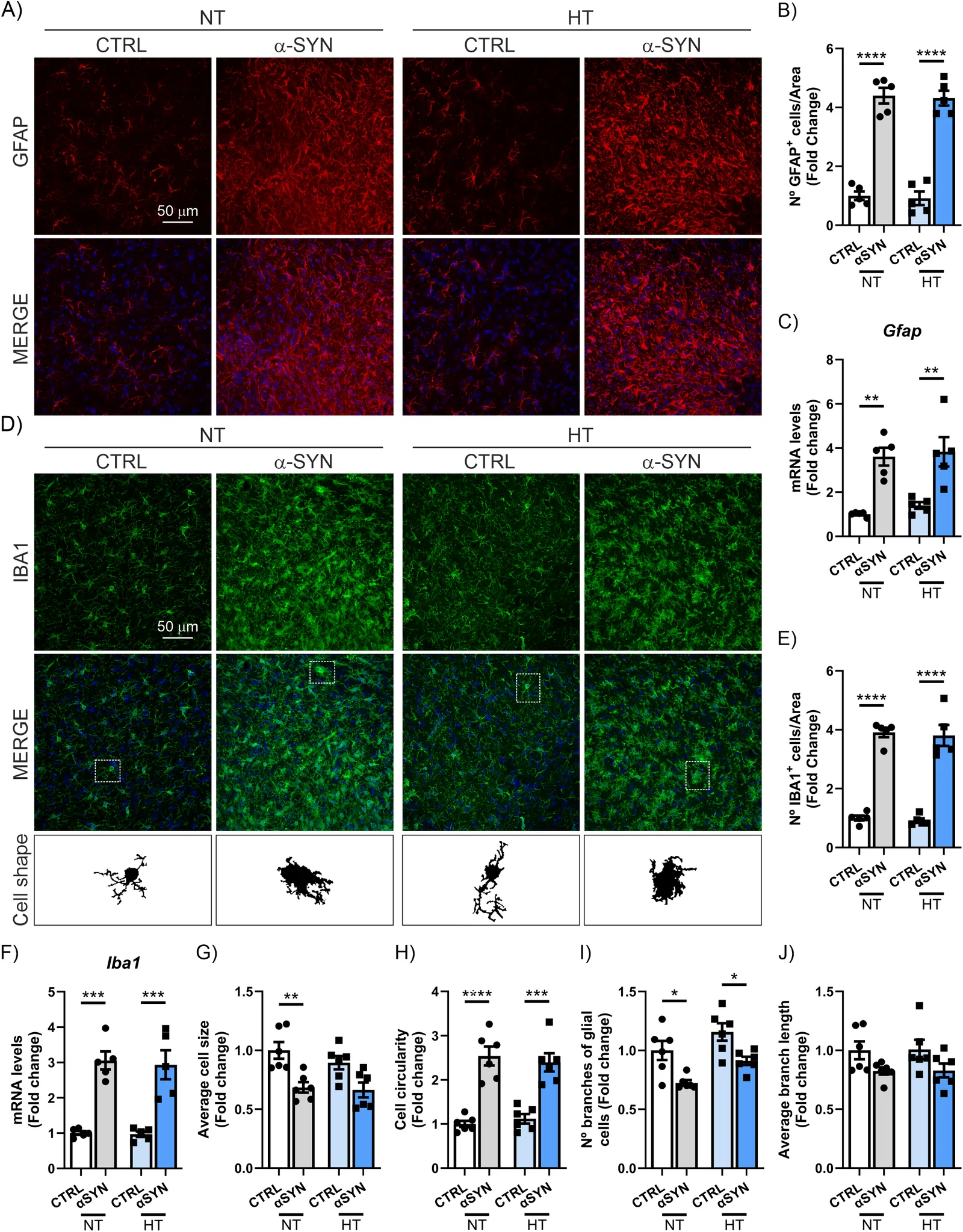
Astrogliosis and microgliosis produced by α-SYN overexpression is not altered by arterial hypertension. **(A)** Representative images of immunofluorescence of GFAP^+^ astrocytes (red) in 30-µm coronal sections of SN. **(B)** Quantification of the number of GFAP^+^ cells in the CTRL and α-SYN overexpressing sides of SN of HT and NT mice. RT-qPCR determination of mRNA levels of **(C)** *Gfap* in the CTRL and α-SYN overexpressing sides of VMB of HT and NT. **(D)** Representative images of immunofluorescence of IBA1^+^ microglial cells (green) in 30-µm coronal sections of SN. On the bottom side of each image, the shape of a selected sample cell (white square) is represented. **(E)** Quantification of the number of IBA1^+^ cells in the CTRL and α-SYN overexpressing sides of SN of HT and NT mice. **(F)** RT-qPCR determination of mRNA levels of *Iba1* in the CTRL and α-SYN overexpressing sides of VMB of HT and NT mice. Morphological analysis of the **(G)** average cell size, **(H)** cell circularity, **(I)** average number of branches and **(J)** the mean length of these branches of glial IBA1^+^ cells. Bars represent the mean of 5–6 samples ± SEM. The asterisks represent the difference in significance: *p<0.05; **p<0.01; ***p<0.001, ****p<0.0001 comparing each group with the control side from NT mice according to two-way ANOVA followed by Bonferroni post-test. Comparisons without asterisks indicate no statistically significant differences (p > 0.05).

### 3.3 Hypertension does not exacerbate α-SYN-induced microgliosis and neuroinflammatory responses

In the context of neuroinflammation, we next investigated whether hypertension could modulate microgliosis, which drives inflammatory responses. First, we performed immunofluorescence staining for IBA1 (ionized calcium-binding adapter molecule 1), a microglial marker, and observed that α-SYN overexpression induced a highly significant increase in the number of IBA1^+^ cells (**Figures 2D, E**), a finding further confirmed by the analysis of *Iba1* mRNA levels (**Figure 2F**). However, hypertension did not appear to alter this microgliotic response. Likewise, morphological analysis of microglia revealed that α-SYN overexpression reduced cell size (**Figure 2G**), increased microglial circularity (**Figure 2H**), and decreased both the number of branches (**Figure 2I**) and their length (**Figure 2J**). Nevertheless, hypertension did not induce changes in any of these parameters. No effects of hypertension were observed in control animals either. The observed microgliosis was attributable to α-SYN overexpression rather than to the AAV injection itself, as no microgliosis was detected following injection of AAV-EGFP (data not shown).

We then performed a more comprehensive analysis of the inflammatory response. We found that *Rela* mRNA levels, encoding the p65 subunit of the NF-κB transcription factor, were increased following α-SYN overexpression, although this effect did not reach statistical significance (**Figure 3A**). Interestingly, hypertension potentiated this increase, resulting in statistically significant differences. In the case of *Il6*, hypertension induced a mild increase in expression under control conditions; however, it did not further enhance the effect triggered by α-SYN overexpression (**Figure 3B**). Finally, we analyzed the expression levels of another pro-inflammatory cytokine, *Tnfa*, as well as *Trem2* and *Tyrobp*, which form a key signaling complex in microglia regulating cell survival, phagocytosis, and responses to neuroinflammation. In all cases, α-SYN overexpression induced a highly significant increase in the mRNA expression of these genes, whereas hypertension did not produce any additional effect (**Figures 3C–E**). Taken together, these results suggest that hypertension does not modify key parameters of microgliosis either under control conditions or in response to α-SYN overexpression.

**Figure 3:**
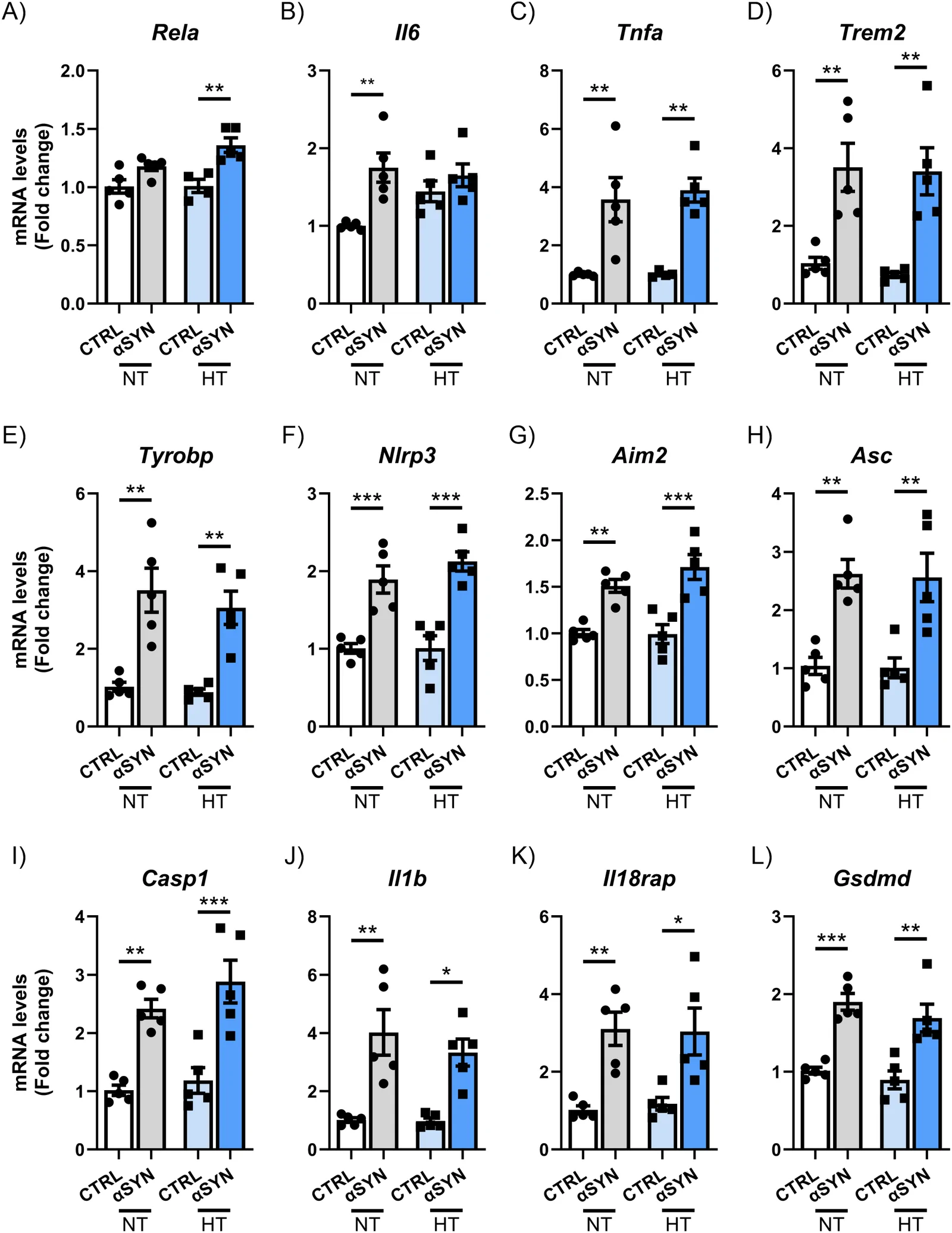
Hypertensive mice have similar α-SYN induced inflammation/pyroptosis than NT mice. RT-qPCR determination of pro-inflammatory markers mRNA levels of **(A)** *Rela*, **(B)** *Il6*, **(C)** *Tnfa*, **(D)** *Trem2* and **(E)** *Tyrobp* in the CTRL and α-SYN overexpressing sides of VMB of HT and NT mice. RT-qPCR determination of pyroptosis markers mRNA levels of **(F)** *Nlrp3*, **(G)** *Aim2*, **(H)** *Asc*, **(I)** *Casp1* **(J)** *Il1b*, **(K)** *Il18rap* and **(L)** *Gsdmd* in the CTRL and α-SYN overexpressing sides of VMB of HT and NT mice. Bars represent the mean of 5–6 samples ± SEM. The asterisks represent the difference in significance: *p<0.05; **p<0.01; ***p<0.001, comparing each group with the control side from NT mice according to two-way ANOVA followed by Bonferroni post-test. Comparisons without asterisks indicate no statistically significant differences (p > 0.05).

### 3.4 Hypertension does not modify α-SYN-induced activation of pyroptosis-related pathways in microglia

Next, we performed an in-depth analysis of the expression of genes involved in pyroptosis, an NF-κB-dependent and highly inflammatory form of programmed cell death initiated by inflammasome activation (41). This multiprotein complex is composed of NLRP3 (NOD-like receptor family pyrin domain-containing 3), the adaptor protein ASC (apoptosis-associated speck-like protein containing a CARD), and pro-CASPASE-1. Upon activation, NLRP3 recruits ASC, which subsequently promotes the cleavage and activation of CASPASE-1. Activated CASPASE-1 then processes GASDERMIN D (GSDMD into NT-GSDMD), leading to membrane pore formation, cell lysis, and the release of pro-inflammatory cytokines such as IL-1β and IL-18. In the context of neurodegenerative diseases, increasing evidence suggests that α-SYN aggregates activate the NLRP3/ASC/CASPASE-1 axis, particularly in microglia, thereby triggering pyroptosis and sustaining chronic neuroinflammation (11, 12). Accordingly, we analyzed the mRNA expression levels of genes associated with this pathway. α-SYN overexpression significantly increased the mRNA levels of *Nlrp3*, *Asc*, *Aim2*, *Casp1*, *Il1b*, *Il18rap*, and *Gsdmd*, indicating activation of pyroptosis-related pathways (**Figures 3F–L**). However, hypertension did not modify the expression of any of these genes under either control conditions or following α-SYN overexpression (**Figures 3F–L**). To further characterize this process in our AAV-α-SYN mouse model of PD, we performed immunofluorescence (IF) analyses using IBA1 as a microglial marker and observed that GSDMD-positive cells corresponded to microglia (**Figure 4A**). We observed that the GSDMD-positive cells were microglia, as corroborated by the colocalization profile analysis (**Figure 4B**). In addition, α-SYN overexpression significantly increased both GSDMD protein levels, assessed by mean fluorescence intensity (**Figure 4C**), and the number of GSDMD^+^ cells (**Figure 4D**). In contrast, hypertension did not produce any detectable effect on these parameters. Although no commercially available NT-GSDMD-specific antibodies validated for immunofluorescence in mouse tissue are currently available, limiting direct confirmation of GSDMD cleavage by IF, our findings demonstrate that GSDMD expression occurs specifically in microglial cells, as it was exclusively detected in IBA1^+^ cells. Taken together, these findings indicate that α-SYN overexpression strongly activates pyroptosis-related pathways in microglia, as evidenced by the induction of inflammasome-associated genes and increased GSDMD expression. However, hypertension did not modify any of these molecular or cellular parameters, suggesting that it does not exacerbate microglial pyroptotic responses either under basal conditions or in the context of α-SYN-driven neuroinflammation.

**Figure 4:**
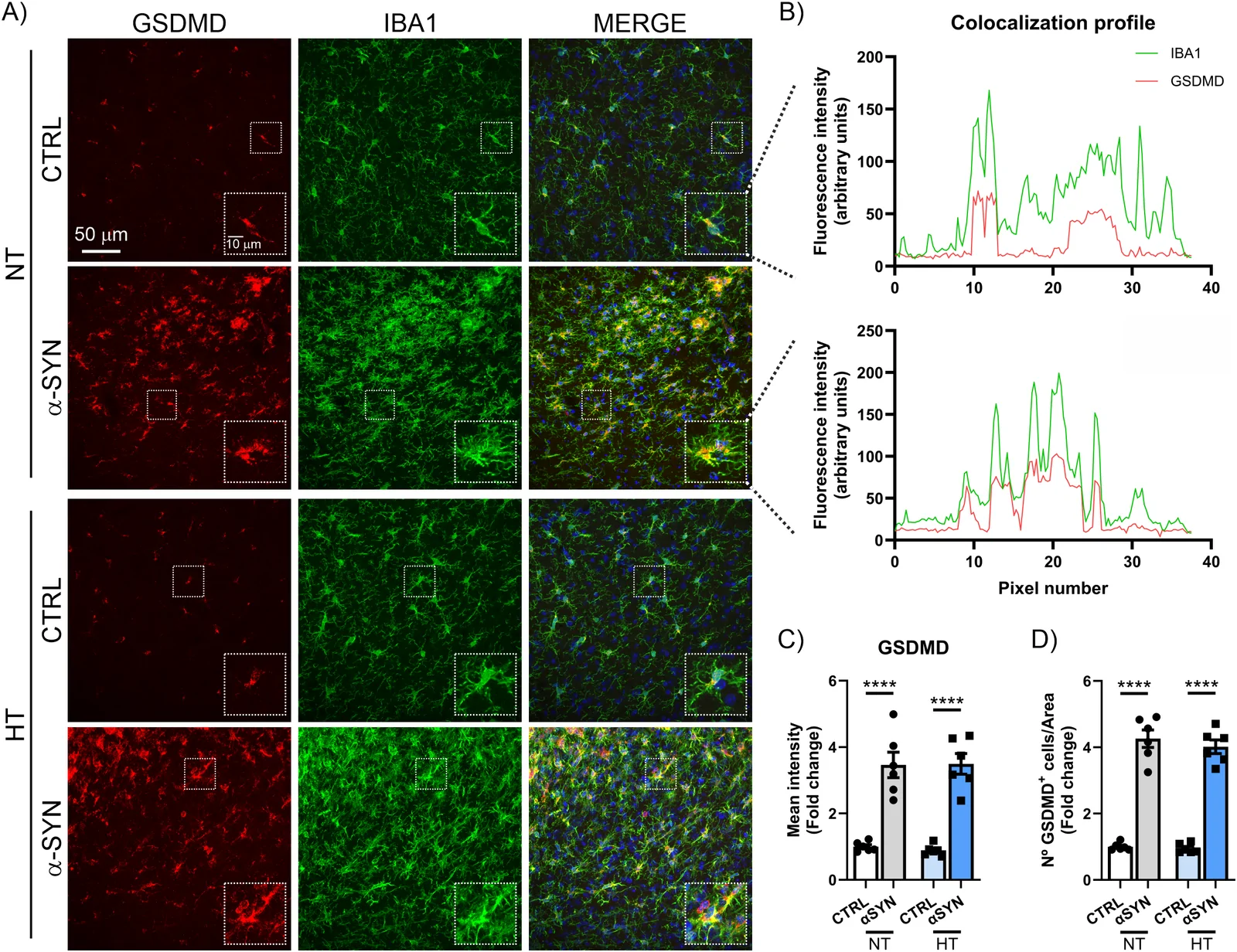
Arterial hypertension does not alter the α-SYN-driven GSDMD-pyroptotic process in microglial cells of the PD mouse model. **(A)** Representative images of GSDMD (red) and IBA1 (green) immunofluorescence in 30-µm coronal sections of the CTRL and α-SYN overexpressing sides of SN of HT and NT mice SN. **(B)** Colocalization profile for CTRL-NT and α-SYN-NT samples Quantification of **(C)** GSDMD fluorescence intensity and **(D)** the number of GSDMD^+^ cells. Bars represent the mean of 5–6 samples ± SEM. The asterisks represent the difference in significance: ****p<0.0001, comparing each group with the control side from NT mice according to two-way ANOVA followed by Bonferroni post-test. Comparisons without asterisks indicate no statistically significant differences (p > 0.05).

## 4 Discussion

The present study investigated whether chronic arterial hypertension exacerbates α-SYN-driven neurodegeneration and neuroinflammation in an AAV-based mouse model of PD. Although epidemiological and experimental evidence suggests that hypertension contributes to brain inflammation and may increase the risk or progression of neurodegenerative disorders (13, 14, 16, 18), our results demonstrate that 3 weeks AngII-induced hypertension did not aggravate nigral dopaminergic degeneration, gliosis, or pyroptosis-related inflammatory pathways triggered by α-SYN overexpression. These findings indicate that, at least under the experimental conditions used here, systemic hypertension is not sufficient to potentiate the early inflammatory and neurodegenerative events induced by α-SYN in the SN.

Several clinical studies have proposed an association between cardiovascular dysfunction and PD, including hypertension as a potential risk factor for disease progression or severity (20, 21, 23). In parallel, chronic hypertension has been associated with neurovascular dysfunction, blood–brain barrier impairment, oxidative stress, and persistent low-grade inflammation in the central nervous system (15, 17, 19). Moreover, morphological alterations of dopaminergic neurons have been reported in spontaneously hypertensive rats, suggesting that elevated blood pressure could sensitize the nigrostriatal pathway to neurodegenerative insults (40).

Based on these observations, we hypothesized that hypertension could amplify α-SYN-induced neurotoxicity. However, despite the robust elevation in arterial pressure achieved after chronic AngII infusion, no increase in dopaminergic neuronal loss was detected in hypertensive mice compared with normotensive controls after AAV-mediated α-SYN overexpression. One possible explanation for the absence of synergistic neurodegeneration is that the inflammatory burden induced by α-SYN overexpression alone may already reach a near-maximal activation state in this acute experimental paradigm. Indeed, α-SYN overexpression produced pronounced microgliosis, astrogliosis, induction of pro-inflammatory cytokines, and activation of inflammasome-associated genes, consistent with previous reports demonstrating that pathological α-SYN is a potent trigger of innate immune activation in PD (28, 29). In this context, hypertension may not provide an additional inflammatory stimulus sufficient to further enhance these already robust responses within the relatively short time frame analyzed. It would be interesting to test whether shorter times of α-SYN administration, where a less pronounced inflammatory response is expected, could differently affect brain inflammation in normotensive and hypertensive mice. Additionally, duration of hypertension in our model was 3 weeks which clearly augment blood pressure and produces cardiovascular damage. However, this is insufficient to clearly induce an inflammatory response in the brain.

Beyond these biological considerations, the experimental design also presented methodological limitations that should be acknowledged. At the experimental endpoint, motor function was intended to be evaluated using the torsion test (29–31), a behavioral readout previously shown to correlate with the extent of dopaminergic injury. However, the presence of the implanted Alzet pumps on the dorsal side of the animals mechanically interfered with rotational movements, making this assay unsuitable under the present conditions.

As mentioned before, astrocytes and microglia are central mediators of neuroinflammatory processes in PD. Activated astrocytes can release cytokines, reactive oxygen species, and inflammatory mediators that contribute to neuronal dysfunction, whereas microglia orchestrate innate immune responses and participate in α-SYN clearance (41). In our study, α-SYN overexpression induced significant increases in GFAP-positive astrocytes and IBA1-positive microglia, accompanied by characteristic morphological changes in microglia associated with activation. However, hypertension did not modify any of these cellular responses. Interestingly, although hypertension slightly increased *Rela* expression, this effect was modest and did not translate into broader amplification of inflammatory cytokines or gliosis markers. These findings suggest that systemic hypertension alone may not be sufficient to alter glial reactivity in the SN during the early stages of α-SYN pathology.

One of the most relevant findings of this work is that hypertension failed to potentiate pyroptosis-related pathways activated by α-SYN. Increasing evidence indicates that inflammasome activation, particularly through the NLRP3 pathway, contributes to PD pathogenesis (7–9). Aggregated α-SYN can activate microglial NLRP3 inflammasomes, leading to CASPASE-1 activation, GSDMD-mediated membrane permeabilization, and release of IL-1β and IL-18, thereby sustaining chronic neuroinflammation and neuronal injury (11, 12). Consistent with these studies, α-SYN overexpression in our model induced marked upregulation of *Nlrp3*, *Asc*, *Casp1*, *Il1b*, *Il18rap*, and *Gsdmd* expression. Furthermore, GSDMD immunoreactivity was selectively localized in IBA1-positive microglial cells, supporting the notion that microglia are the primary cellular substrate of pyroptotic signaling in this model. Nevertheless, hypertension did not modify any of these molecular or cellular parameters, indicating that elevated arterial pressure does not amplify inflammasome-dependent neuroinflammatory responses during early α-SYN pathology.

Increasing evidence indicates that RAAS may participate in PD pathogenesis. Although retrospective clinical studies have yielded somewhat heterogeneous results, several reports suggest that patients receiving RAAS inhibitors, including angiotensin-converting enzyme inhibitors and AT1 receptor blockers, may exhibit a lower risk of developing PD or experience a slower clinical progression (42–45). Supporting a more direct role of RAAS signaling within the nigrostriatal system, recent single-cell transcriptomic analyses identified a subpopulation of dopaminergic neurons in the ventral tier of the SNpc characterized by high AT1 receptor expression, which are among the neurons most vulnerable to degeneration in PD (46). Unpublished data from our lab supports this observation. Together, these observations suggest that excessive AT1 receptor signaling may contribute to neuronal susceptibility through mechanisms beyond or additional to its systemic effects on arterial pressure, including oxidative stress and neuroinflammatory responses. Although our results indicate that short-term AngII-induced hypertension does not exacerbate α-SYN-mediated pathology, they do not exclude the possibility that direct modulation of AT1 receptor signaling could influence disease progression. Indeed, selective inhibition of AT1 receptor remains an attractive therapeutic strategy that deserves further investigation in experimental models of α-SYN pathology.

The lack of interaction between hypertension and α-SYN pathology may also relate to the duration and severity of the hypertensive insult. Although AngII infusion produced a clear hypertensive phenotype, the experimental window was relatively short and may reflect an early disease stage. Chronic hypertension over longer periods could induce cumulative cerebrovascular damage, endothelial dysfunction, or blood–brain barrier alterations that eventually interact with α-SYN pathology. In this sense, other studies have used AngII infusion for up to 3 months using lower doses of AngII (47, 48). Future studies employing aged animals, longer hypertension paradigms, or models displaying more severe vascular impairment may therefore reveal synergistic effects not detected here. Additionally, behavioral evaluation was limited because the implanted osmotic pumps interfered with rotational motor testing. Therefore, subtle functional consequences of hypertension could not be fully assessed in the present study.

Despite these limitations, our findings provide important insight into the relationship between vascular risk factors and α-SYN-mediated neurodegeneration. The data suggest that hypertension alone does not necessarily exacerbate early α-SYN-induced neuroinflammatory and pyroptotic responses in the SN. These results refine our understanding of the contribution of cardiovascular comorbidities to PD pathogenesis and indicate that additional mechanisms, such as aging, cerebrovascular injury, metabolic dysfunction, or prolonged systemic inflammation, may be required for hypertension to significantly influence α-SYN-driven neurodegeneration.

Overall, this study demonstrates that three-weeks AngII-induced hypertension does not aggravate dopaminergic neuronal loss, gliosis, inflammatory gene expression, or microglial pyroptosis induced by α-SYN overexpression in the SN. Our findings support the concept that α-SYN pathology itself is the dominant driver of neuroinflammatory activation in this model and suggest that hypertension, in isolation, is insufficient to potentiate these pathogenic processes during the early stages of disease progression.

## Data Availability

The original contributions presented in the study are included in the article/**Supplementary Material**. Further inquiries can be directed to the corresponding author.
