# Supplementary material for: Angiotensin II-induced hypertension does not exacerbate α-SYNUCLEIN pathology in a mouse model of Parkinson’s disease
Source: Front Immunol. 2026 Sep 22;17:1894579. doi: 10.3389/fimmu.2026.1894579 (PMC13651558; doi:10.3389/fimmu.2026.1894579)
Supplement: Supplementary file 1 [file Table1.docx]

**Supplementary Table 1.** List of antibodies used in this study.

| Antibody | Source | Catalog Number | Dilution |
| --- | --- | --- | --- |
| α-SYNUCLEIN (α-SYN) | Millipore | AB5038P | 1:2000 IHC |
| Alexa Fluor 488 donkey anti-rabbit IgG | Thermo Fisher Scientific | A21206 | 1:500 IF |
| Alexa Fluor 546 donkey anti-mouse IgG | Thermo Fisher Scientific | A31570 | 1:500 IF |
| Alexa Fluor 555 donkey anti-rabbit IgG | Thermo Fisher Scientific | A31572 | 1:500 IF |
| Alexa Fluor 647 goat anti-guinea pig IgG | Thermo Fisher Scientific | A21450 | 1:500 IF |
| Biotinylated anti-mouse IgG (H+L) | Vector Laboratories | BA-9200 | 1:500 IHQ |
| Biotinylated anti-rabbit IgG (H+L) | Vector Laboratories | BA-1000 | 1:500 IHQ |
| GASDERMIN D (GSDMD) | Abcam | AB219800-1001 | 1:500 IF |
| GFAP | Sigma-Aldrich | G3893 | 1:400 IF |
| IBA1 | Wako Chemicals | 019-19741 | 1:500 IF |
| IBA1 | Synaptic Systems | HS-234 308 | 1:1000 IF |
| TH | MERCK | MAB5280 | 1:500 IF / IHQ |
