# Supplementary material for: Angiotensin II-induced hypertension does not exacerbate α-SYNUCLEIN pathology in a mouse model of Parkinson’s disease
Source: Front Immunol. 2026 Sep 22;17:1894579. doi: 10.3389/fimmu.2026.1894579 (PMC13651558; doi:10.3389/fimmu.2026.1894579)
Supplement: Supplementary file 2 [file Table2.docx]

**Supplementary Table 2.** List of primers used in this study.

| **Gene Product** | **Forward Primer (5’-3’)** | **Reverse Primer (5’-3’)** |
| --- | --- | --- |
| *Actb* | TCCTTCCTGGGCATGGAG | AGGAGGAGCAATGATCTTGATCTT |
| *Aim2* | GTCACCAGTTCCTCAGTTGTG | CACCTCCATTGTCCCTGTTTTAT |
| *Asc* | AGAGTACAGCCAGAACAGGA | GATGGAACAAAGCTGAAGAG |
| *Casp1* | GGAGAGAAACAAGGAGTGGTGT | ACTCAATGAAAAGTGAGCCCCT |
| *Gapdh* | CGACTTCAACAGCAACTCCCACTCTTCC | TGGGTGGTCCAGGGTTTCTTACTCCTT |
| *Gfap* | TCCTGGAACAGCAAAACAAG | CAGCCTCAGGTTGGTTTCAT |
| *Gsdmd* | TTCAGGGCAGAGTGATGTTGT | TCATCCCCACGACTCCGAA |
| *Iba1* | GTCCTTGAAGCGAATGCTGG | CATTCTCAAGATGGCAGATC |
| *Il18rap* | GTCCGAGCTGTGGTTAAAGTG | AGGCTTTCCAAGCTCTACGTC |
| *Il1b* | CTGGTGTGTGACGTTCCCATTA | CCGACAGCACGAGGCTTT |
| *Il6* | CCTACCCCAATTTCCAATGCT | TATTTTCTGACCACAGTGAGGAATG |
| *Nlrp3* | CCCTTGGAGACACAGGACTC | GAGGCTGCAGTTGTCTAATTCC |
| *Rela* | GGCCTCATCCACATGAACTT | CACTGTCACCTGGAAGCAGA |
| *S100b* | GTTACTCGGACACTGAAGCCA | GGAAGTGAGAGAGCTCGTTGT |
| *SNCA* | AGGCTCCAAAACCAAGGAGG | TGCTCCCTCCACTGTCTTCT |
| *Tbp* | CTGGCGGTTTGGCTAGGTTT | TGAGCATAAGGTGGAAGGCTG |
| *Tnfa* | CATCTTCTCAAAATTCGAGTGACAA | TGGGAGTAGACAAGGTACAACCC |
| *Trem2* | TGCAGAAAGTACTGGTGGAGGT | CTAGAGGTGACCCACAGGATGAAA |
| *Tyrobp* | GAGGTCAAGGGACAGCGGAA | GCTTGTTGAGTAGGCATCTGG |
